# A Representative Analysis of Nonparticipation in Workplace Health Promotion in Germany Using Multivariable Methods

**DOI:** 10.3389/ijph.2024.1607261

**Published:** 2024-12-05

**Authors:** Birgit Pache, Britta Herbig, Dennis Nowak, Christian Janssen

**Affiliations:** ^1^ Munich University of Applied Sciences, Munich, Germany; ^2^ Institute and Clinic for Occupational, Social and Environmental Medicine, LMU University Hospital, LMU Munich, Munich, Germany; ^3^ Society, Health and Education Research Center/SHE:RC, Munich University of Applied Sciences, Munich, Germany

**Keywords:** work health promotion, nonparticipation, determinants, logistic regression, representative sample

## Abstract

**Objectives:**

Studies have identified sociodemographic and socioeconomic factors that promote participation in workplace health promotion activities. The present study therefore focuses on what influences nonparticipation within a representative sample of the German population.

**Methods:**

In the analysis of possible factors influencing nonparticipation, company characteristics are accounted for in addition to sociodemographic and health behaviour-related variables. The data used for the analysis are from the GEDA study 2014/2015-EHIS of the Robert Koch Institute in Berlin.

**Results:**

Age largely increased the probability of nonparticipation (OR: between 1.30 and 1.92, p: between <0.001 and 0.033). Other possible influencing factors, such as weight, smoking status, alcohol consumption, exercise status and diet, seemed to play a rather minor role in the present analysis. Self-rated belonging to a certain socioeconomic status group also had a significant influence (OR: 0.76, p: <0.001).

**Conclusion:**

The influencing factors seem to be of a sociodemographic and socioeconomic nature. These determinants should be accounted for to reduce nonparticipation. However, a comparison with current or longitudinal data would be needed to prove to what extent the results are still valid or influenced by a cohort effect.

## Introduction

An increasing number of companies advertise workplace health promotion (WHP) in their job offers. According to the Ottawa Charta, developed by the WHO in 1986, living and working conditions should be made not only safe and invigorating but also satisfying and enjoyable through health-promoting measures [1]. The Luxembourg Declaration understands WHP as an interaction between employers, employees and society to improve health and wellbeing at work [2]. The aim of WHP is to improve work organisation and conditions, promote active employee participation and strengthen personal competencies [2]. This is also the difference to health prevention: prevention aims to avoid illness and the resulting damage. Health promotion supports people’s health resources [3, 4].

Several publications show that companies have recognised and successfully responded to the need for WHP. This can reduce absenteeism and thus sickness costs, with a positive effect on the company’s success, the so-called return on investment (ROI) [5–10]. However, an analysis of statistical data on the prevalence of and participation in WHP measures shows that only 3.9% of all employees and only 0.5% of companies implement WHP measures [11–13]. These figures refer only to companies that have implemented WHP measures with the help of health insurance funds. Considering other providers, the total number is probably greater [14–16].

Although the importance and success of WHP have already been presented many times, the question of why not all employees are reached or take part in WHP has arisen. What are the reasons for this, and how is it related to other characteristics of individuals or structures? In the research field, greater focus is placed on successful implementation and on the number of participants, while less attention is given to those who do not participate in the offered WHP. Previous studies, which mainly examined specific institutions or organizations, identified possible barriers to participation [8, 17, 18]. A recent large-scale study by Nöhammer et al. [19] identified employee perceived barriers to WHP use by asking specifically about predetermined barriers, while this study attempts to find other more general and possibly unknown influencing factors that may explain why an employee does not participate in a health promotion programme offered by the employer.

## Methods

For the current analysis, data from the survey “German Health Update” (GEDA 2014/2015-EHIS), collected on a regular basis by the Robert Koch Institute (RKI), were used. Based on a sample survey of residents’ registration offices (using a two-stage stratified cluster), persons aged 18 years and older with permanent residence in Germany were randomly selected from November 2014 to July 2015. The survey was conducted using a questionnaire which was made available online and in paper-and-pencil form and had to be completed by the respondents themselves. A weighting factor was used to correct for deviating population structures in the sample in comparison to the German population (as at 31/12/2014) [9]. The GEDA 2014/2015-EHIS is the only and most recent representative data set for participation or nonparticipation in different specified WHP measures in Germany. In the subsequent 2019/2020 survey wave this topic was no longer included. A somewhat older large population-based study from 2012 (BIBB/BAuA labour force survey) only asks in general terms whether WHP measures were offered in the company in the last 2 years, without specifying them, and whether people had participated or not [20].

For better comparability with previous results from other studies, the following procedure was largely based on Hermann et al. (2021) and Ludwig et al. (2020) [21, 22]. In the present analysis, the age group was restricted to individuals between 18 and 64 years. Only those who stated that one or more WHP measures had been offered in their company in the last 12 months were included. A total of 10 measures were included, as follows: “Did your company/enterprise offer (…) in the last 12 months?” With possible answers of “yes,” “no” and “do not know.” If the answer to this question was “yes,” the following question was asked: “Have you taken advantage of this offer?” The answer options were “yes” and “no.” Two questions relating to WHP were asked in the GEDA-survey. There are no questions about the intensity or frequency of participation. Only respondents who answered the question “Which life situation applies to you predominantly at present?” With full-time, part-time, semiretired or marginally employed (e.g., mini-jobs) were included. For the question “What is your main professional position in your main occupation?,” apprentices were considered in addition to employees, workers and civil servants (including trainees), as this group is often disregarded in publications. All individuals without access to WHP measures, such as non-employed people, self-employed individuals or housewives/househusbands, were excluded.

In contrast to Hermann et al. (2021) and Ludwig et al. (2020) [21, 22], the aim of this study is to include all of the WHP measures as much as possible and not to restrict the survey to just one or three measures. In the end, eight measures were examined in detail. Because of the small number of respondents to smoking cessation offers and staff surveys, no meaningful results could be obtained, and these items were not considered in detail.

**FIGURE 1:**
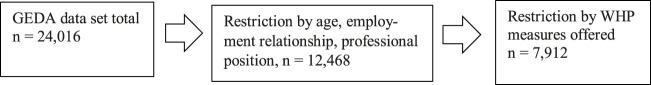
Step-by-step containment of the data set (GEDA study 2014/2015-EHIS, Berlin 2017).

A total of 7,912 of the original 24,016 respondents were considered for the present analysis. In the WHP measures, the number fluctuates from 1,170 to 3,648 according to the different measures offered. Figure 1 shows the step-by-step containment of the data set.

With the present data set, this study sought to identify as many factors influencing nonparticipation in WHP offers as possible at the population level. The selection of factors that could influence WHP measures was based on previous publications and expanded to include other possible factors. Sociodemographic factors (age, gender and socioeconomic status (SES)), subjective health status and health awareness, as well as company characteristics (company size, industry affiliation, working hours and occupational status), have been shown to influence participation in previous studies. Social support, weight, smoking, alcohol, exercise and nutrition have been surveyed less frequently or not at all [6, 20–23]. To determine whether the already known influencing factors also play a role in nonparticipation, these factors were included in the analysis and supplemented by less known factors (e.g., subjective social status and life satisfaction), which were also surveyed in the GEDA study. Table 1 shows the general descriptive values of all analysis variables, which are explained further below.

**TABLE 1:** Overview of the central characteristics of the analysis sample (employed persons aged 18–64) in absolute frequency and percentage based on data from the Robert Koch Institute (GEDA study 2014/2015-EHIS, Berlin 2017, n = 7,912).

	Total n			%
Gender				
Men	3,720			47.0
Women	4,192			53.0
Age				
18–29 years	1,331			16.8
30–44 years	2,674			33.8
45–64 years	3,904			49.4
Socioeconomic status (SES)				
Low	629			7.9
Middle	4,257			53.8
High	3,025			38.2
Subjective social status				
Lower-middle	3,148			39.8
Higher-upper	4,683			59.2
Missing	81			1.0
Subjective Health Status				
Poor-moderate	1,509			19.1
Good-very good	6,381			80.6
Missing	22			0.3
Attention to health				
Less strong-not at all	4,205			53.1
Strong-very strong	3,676			46.5
Missing	31			0.4
BMI				
Underweight	129			1.6
Normal weight	3,955			50.0
Overweight	2,610			33.0
Obesity	1,168			14.8
Missing	50			0.6
Smoking				
Yes	2008			25.4
No	5,897			74.5
Missing	7			0.1
Alcohol				
Yes	6,295			79.6
No	1,608			20.3
Missing	9			0.1
Sport per week				
No - little sport	6,495			82.1
Much - daily sport	1,384			17.5
Missing	33			0.4
Nutrition					
Unhealthy nutrition		2,976	37.6		
Healthy nutrition		4,936	62.4		
Number of employees in the company					
0–10		640	8.1		
11–19		660	8.3		
20–49		887	11.2		
50+		5,680	71.8		
Missing		45	0.6		
Business sector					
Manufacturing and processing industries		1799	22.7		
Service sector		1826	23.1		
Public service		3,371	42.6		
Others		251	3.2		
Missing		665	8.4		
Professional position					
Employee		5,851	74.0		
Worker		862	10.9		
Officer		928	11.7		
Trainee		271	3.4		
Working hours					
Full-time		5,865	74.1		
Part-time		1763	22.3		
Marginally employed		197	2.5		
Partial retirement		87	1.1		
Social support					
Low		1,071	13.5		
Middle		4,404	55.7		
High		2,373	30.0		
Missing		65	0.8		
Life satisfaction					
Not at all-rather		779	9.8		
Satisfied-completely satisfied		7,114	89.9		
Missing		19	0.2		

### Sociodemographic Factors

Sociodemographic factors include gender (male/female), age (divided into three age groups by GEDA: 18–29, 30–44 and 45–64) [21, 22] and socioeconomic status (SES). The basis for calculating SES is schooling and vocational training, occupational status and weighted net household income. In the GEDA study, SES is calculated as an index variable based on predefined scores from 1 to 7 assigned to the individual indicators education, occupational status and net equivalised income. Higher scores indicate higher education, status, and income, respectively. The equally weighted indicator scores are summed up and sum score distribution is used to define three status groups: the low- and high-status groups each include 20% of the population, and 60% of the population is in the middle group [24]. The sample used deviates from these proportions to the disadvantage of low SES. Uneven distribution is a well-known problem in surveys [25]. There are newer approaches than SES (e.g., programme characteristics) as predictors of participation in health measures, but these cannot be used in population-representative studies across many different measures and SES retains its significance even when compared with other data. Furthermore, a lot of studies still prove existing health differences by SES [26].

### Subjective Health and Social Factors

Among other factors, the subjective state of health was accounted for. For the question “What is your state of health in general?,” there were five answer categories, ranging from “very good” to “very bad.” In addition to the different cell assignments, these categories were summarised as “bad - moderate” and “well - very well” according to Ludwig et al. (2020) [22] for better comparability.

The same procedure was used for the question “How much do you generally pay attention to your health?” with the categories “not at all - little attention” and “much - very much.”

Another influencing factor is subjective social status (SSS) measured with the MacArthur scale [27]. Here, respondents were asked to assign their perceived position in society on a scale of 1–10: 10 for the highest education, highest income and best job and 1 for lowest education, low income and poor or no job. To keep the calculation model small, the SSS data were combined into two categories, with values of 1-5 evaluated as “lower to middle stratum” and values of 6–10 evaluated as “upper to highest stratum.”

Other factors proven to influence health are often omitted from the consideration of health promotion measures: weight, smoking, alcohol, exercise and nutrition. For the indicator “weight,” the variable used in the data set was the BMI with the categories “underweight” (BMI <18.5), “normal weight” (18.5 ≤ BMI <25), “overweight” (25 ≤ BMI <30) or “obese” (BMI ≥30). The four answer options on smoking behaviour from “yes, daily” to “never smoked” were combined into the categories “smoker” and “nonsmoker.” The same procedure was used for alcohol consumption, with six answer options (daily to no consumption), and for sport activity per week (from 0 = no sport or sport activity 1 time per week to 7 = daily sport activity). The nutrition indicator was formed by the variables of fruit and vegetable consumption in an additive index and then divided into two groups by a mean split in the categories of “unhealthy” and “healthy nutrition.”

### Company Characteristics

Three known aspects were accounted for here. The first is the size of the company, which is generally also an important factor in the introduction and implementation of WHP. The following subdivisions were available for selection: 1–10, 11–19, 20–49 and 50+ employees. Second is the occupational sector, regarding which the dataset contained a total of 21 response categories. As it is not necessarily the exact occupational field that is important but rather the economic sector and as there is no common categorization in other studies [6, 20, 22, 28], the responses were grouped into four basic categories: manufacturing/processing, services, public service/healthcare/social services/administration, and other. The third characteristic is the individual’s position in the profession, with the following potential responses being included: employees, workers, civil servants (including trainees) and apprentices.

### Other Characteristics

Perceived social support and general life satisfaction were examined as further potential influencing factors. Perceived social support, (here in relation to friends, family and neighbours) was surveyed using the Oslo 3-Item Social Support Scale [29], and the answers were divided into “low,” “middle” and “high.” For general life satisfaction (“Generally, how satisfied are you with your life overall?”), the original 10 response categories were again combined into two categories: “not at all - moderately” and “highly - very highly.”

The analysis was carried out using logistic regression. Several factors that may influence dichotomous variables were examined. The model requirements of no outliers (standardised residuals: −3 and 3) and no multicollinearity (VIF values below 5) were checked. Since there were no metric factors, there was no need to check the linearity of the logit model.

## Results

Table 2 first shows, listed by frequency of offer, how many respondents were offered WHP measures and their participation and nonparticipation broken down by age, gender and SES.

**TABLE 2:** Descriptive statistics of the dependent variables according to the frequency of the workplace health promotion measures of healthy lunch, back health, company sport, stress management, information/consulting nutrition, help against bullying, financial allowance, discussion/working group in terms of age, gender and socioeconomic status (Calculated for all respondents in employment based on data from the GEDA study 2014/2015-EHIS, Berlin 2017, n = 12,468).

		General		Age			Gender		SES		
				18–29 Years	30–44 Years	45–64 Years	Men	Women	Low	Middle	High
Healthy lunch	Offered	3.972 (31.9%)	Yes	691 (35.9%)	1.345 (33.9%)	1.936 (33.1%)	2.037 (38.3%)	1.935 (30.1%)	280 (20.9%)	2.060 (31.1%)	1.632 (42.7%)
			No	1.234 (64.1%)	2.619 (66.1%)	3.918 (66.9%)	3.275 (61.7%)	4.496 (69.9%)	1.057 (79.1%)	4.520 (68.7%)	2.191 (57.3%)
	Participation	2.524 (20.2%)	Yes	477 (71.6%)	893 (68.1%)	1.154 (61.0%)	1.324 (66.4%)	1.200 (64.0%)	174 (64.7%)	1.206 (60.2%)	1.144 (65.2%)
			No	189 (28.4%)	419 (31.9%)	737 (39.0%)	671 (33.6%)	674 (36.0%)	95 (35.3%)	796 (39,8%)	454 (28.4%)
Back health	Offered	3.222 (25.8%)	Yes	490 (28.8%)	1.110 (29.8%)	1.622 (29.0%)	1.666 (33.8%)	1.556 (25.5%)	194 (15.3%)	1.705 (27.5%)	1.323 (37.1%)
			No	1.213 (71.2%)	2.618 (70.2%)	3,980 (71.0%)	3.259 (66.2%)	4,552 (74.5%)	1.076 (84.7%)	4.490 (72.5%)	2.242 (62.9%)
	Participation	696 (5.6%)	Yes	106 (21.9%)	236 (21.5%)	354 (22.2%)	304 (18.4%)	392 (25.7%)	51 (27.6%)	391 (23.3%)	254 (19.4%)
			No	377 (78. 1%)	860 (78.5%)	1.239 (77.8%)	1.344 (81.6%)	1.132 (74.3%)	134 (72.4%)	1.290 (76.7%)	1.052 (80.6%)
Company sport	Offered	2.900 (23.3%)	Yes	479 (26.7%)	1.074 (28.1%)	1.347 (23.8%)	1.605 (31.5%)	1.295 (20.9%)	139 (10.9%)	1.410 (22.3%)	1.351 (36.6%)
			No	1.316 (73.3%)	2.751 (71.9%)	4.317 (76.2%)	3.491 (68.5%)	4.893 (79.1%)	1.137 (89.1%)	4,905 (77.7%)	2,339 (63.4%)
	Participation	732 (5.9%)	Yes	164 (34.9%)	270 (25.7%)	298 (22.6%)	454 (28.9%)	278 (21.9%)	38 (28.4%)	365 (26.2%)	329 (25.8%)
			No	306 (65.1%)	782 (74.3%)	1.019 (77.4%)	1.118 (71.1%)	989 (78.1%)	96 (71.6%)	1.016 (73.6%)	995 (75.2%)
Stress management	Offered	2.787 (22.4%)	Yes	422 (24.0%)	1.009 (26.7%)	1.356 (24.1%)	1.328 (26.8%)	1.459 (23.5%)	123 (9.7%)	1.332 (21.1%)	1.332 (37.0%)
			No	1.340 (76.0%)	2.773 (73.3%)	4.263 (75.9%)	3.626 (73.2%)	4.750 (76.5%)	1.144 (90.3%)	4.963 (78.8%)	2.266 (63.0%)
	Participation	824 (6.6%)	Yes	138 (33.7%)	262 (26.5%)	424 (32.2%)	324 (24.9%)	500 (35.5%)	48 (40.3%)	406 (31.5%)	370 (28.4%)
			No	272 (66.3%)	725 (73.5%)	892 (67.8%)	979 (75.1%)	910 (64.5%)	71 (59.7%)	883 (68.5%)	935 (71.6%)
Info/Consulting nutrition	Offered	2.638 (21.2%)	Yes	383 (21.8%)	881 (23.5%)	1.374 (24.3%)	1.333 (26.8%)	1.305 (21.1%)	171 (13.3%)	1.382 (22.0%)	1.085 (30.3%)
			No	1.373 (78.2%)	2.868 (76.5%)	4.272 (75.7%)	3.643 (73.2%)	4.870 (78.9%)	1.118 (86.7%)	4.895 (78.0%)	2.497 (69.7%)
	Participation	1.090 (8.7%)	Yes	160 (44.2%)	313 (37.0%)	617 (46.8%)	483 (37.3%)	607 (49.3%)	74 (45.7%)	572 (43.4%)	444 (42.5%)
			No	202 (55.8%)	533 (63.0%)	700 (53.2%)	812 (62.7%)	623 (50.7%)	88 (54.3%)	746 (56.6%)	601 (57.5%)
Help against Bullying	Offered	2.444 (19.6%)	Yes	340 (20.4%)	802 (22.3%)	1.302 (23.8%)	1.242 (26.1%)	1.202 (20.2%)	152 (12.2%)	1.185 (19.6%)	1.107 (32.2%)
			No	1.327 (79.6%)	2.791 (77.7%)	4.164 (76.2%)	3.521 (73.9%)	4.761 (79.8%)	1.094 (87.8%)	4.856 (80.4%)	2.329 (67.8%)
	Participation	399 (3.2%)	Yes	82 (25.5%)	119 (15.5%)	198 (15.9%)	145 (12.0%)	254 (22.5%)	47 (33.6%)	211 (18.7%)	141 (13.2%)
			No	239 (74.5%)	649 (84.5%)	1.050 (84.1%)	1.065 (88.0%)	873 (77.5%)	93 (66.4%)	919 (81.3%)	926 (86.8%)
Financial allowance	Offered	1.929 (15.5%)	Yes	313 (18.4%)	677 (18.5%)	939 (17.2%)	961 (20.1%)	968 (16.0%)	128 (10.3%)	1.026 (16.8%)	774 (22.4%)
			No	1.391 (81.6%)	2.975 (81.5%)	4.521 (82.8%)	3.816 (79.9%)	5.071 (84.0%)	1.115 (89.7%)	5.088 (83.2%)	2.682 (77.6%)
	Participation	567 (4.5%)	Yes	108 (35.3%)	188 (28.2%)	271 (29.7%)	251 (26.5%)	316 (33.7%)	43 (34.7%)	300 (29.9%)	224 (29.6%)
			No	198 (64.7%)	479 (71.8%)	642 (70.3%)	697 (73,5%)	622 (66.3%)	81 (65.3%)	703 (70.1%)	534 (70.4%)
Discussion/Working group	Offered	1.293 (10.4%)	Yes	194 (11.6%)	434 (12.1%)	665 (12.3%)	696 (14.7%)	597 (10.1%)	103 (8.2%)	675 (11.2%)	515 (15.4%)
			No	1.474 (88.4%)	3.147 (87.9%)	4.736 (87.7%)	4.028 (85.3%)	5.329 (89.9%)	1.156 (91.8%)	5.369 (88.8%)	2.829 (84.6%)
	Participation	467 (3.7%)	Yes	69 (36.5%)	137 (33.2%)	261 (40.5%)	224 (33.0%)	243 (42.7%)	46 (46.5%)	272 (42.0%)	149 (29.7%)
			No	120 (63.5%)	276 (66.8%)	384 (59.5%)	454 (67.0%)	326 (57.3%)	53 (53.5%)	375 (58.0%)	352 (70.3%)

At almost 32%, a healthy lunch was offered most frequently. This is also the measure with the highest participation rate across the different groups but also illustrates that nonparticipation is predominating. Interestingly, fewer measures were consistently offered for the low SES group, but the percentage of participants from this group was greater than that of the other groups for each measure. This was also almost universally the case for women. There was no consistent trend regarding age.

Table 3 shows how many employees were offered at least one WHP measure, the distribution in terms of company size and sector and the participation in the measures offered.

**TABLE 3:** Total workplace health promotion measures offered (healthy lunch, back health, company sport, stress management, information/consulting on nutrition, help against bullying, financial allowance, discussion/working group) presented according to frequency in general (n = 7,912) and regarding the number of people in the company (n = 6,972) and the business sector (n = 6,465) based on data from the GEDA study 2014/2015-EHIS, Berlin 2017.

	General	Number of persons in the company				Business sector			
	Frequency (%)	Up to 10	11–19	20–49	50+	Manufacturing and processing industries	Service sector	Public service	Others
One offer	2,137 (27.0%)	250 (50.8%)	260 (48.0%)	299 (41.8%)	1,314 (25.2%)	434 (27.0%)	499 (32.5%)	898 (29.0%)	94 (42.7%)
Two offers	1,346 (17.0%)	122 (24.8%)	126 (23.2%)	170 (23.8%)	981 (17.6%)	270 (16.8%)	286 (18.6%)	628 (20.3%)	40 (18.2%)
Three offers	1,088 (13.8%)	52 (10.6%)	57 (10.5%)	112 (15.7%)	859 (16.4%)	243 (15.1%)	196 (12. 8%)	541 (17.5%)	30 (13.6%)
Four offers	808 (10.2%)	27 (5.5%)	35 (6.5%)	68 (9.5%)	671 (12.8%)	171 (10.6%)	177 (11.5%)	384 (12.4%	27 (12.3%)
Five offers	650 (8.2%)	21 (4.3%)	33 (6.1%)	39 (5.5%)	555 (10.6%)	150 (9.3%)	152 (9.9%)	304 (9.8%)	10 (4.5%)
Six offers	488 (6.2%)	9 (1.8%)	18 (3.3%)	14 (2.0%)	445 (7.0%)	135 (8.4%)	116 (7.5%)	201 (6.5%)	12 (5.5%)
Seven offers	302 (3.8%)	7 (1.4%)	8 (1.5%)	8 (1.1%)	279 (5.3%)	121 (7.5%)	69 (4.5%)	90 (2.9%)	3 (1.4%)
Eight offers	196 (2.5%)	4 (0.8%)	5 (0.9%)	5 (0.7%)	182 (2.8%)	85 (5.3%)	42 (2.7%)	53 (1.7%)	4 (1.8%)
total	7,015	492	542	715	5,223	1,609	1,537	3,099	220

Twenty-seven percent of all employees were offered at least one measure. Three or more measures tended to be offered to employees in large companies with more than 50 employees. In this analysis, measures were most frequently offered in the service sector.

If only one measure was offered, just over half of the respondents took part. If three or more measures were offered, the majority participated in at least one of them (Table 4).

**TABLE 4:** Frequency of workplace health promotion measures (healthy lunch, back health, company sport, stress management, information/consulting on nutrition, help against bullying, financial allowance, discussion/working group) and the participation rate based on data from the GEDA study 2014/2015-EHIS, Berlin 2017.

SUM participation										
SUM offers	0	1	2	3	4	5	6	7	8	total
1	949 (48.4%)	1,012 (51.6%)								1961 (100%)
2	538 (40.7%)	477 (36.1%)	307 (23.2%)							1,322 (100%)
3	335 (31.2%)	379 (35.3%)	246 (22.9%)	113 (10.5%)						1,073 (100%)
4	208 (26.0%)	294 (36.8%)	169 (21.2%)	83 (10.4%)	45 (5.6%)					799 (100%)
5	172 (26.5%)	214 (32.9%)	127 (19.5%)	69 (10.6%)	41 (6.3%)	27 (4.2%)				650 (100%)
6	101 (20.7%)	151 (30.9%)	103 (21.1%)	70 (14.3%)	41 (8.4%)	15 (3.1%)	7 (1.4%)			488 (100%)
7	49 (16.2%)	76 (25.2%)	75 (24.8%)	48 (15.9%)	26 (8.6%)	17 (5.6%)	3 (1.0%)	8 (2.6%)		302 (100%)
8	22 (11.2%)	50 (25.5%)	40 (20.4%)	40 (20.4%)	22 (11.2%)	9 (4.6%)	7 (3.6%)	3 (1.5%)	3 (1.5%)	169 (100%)

The results of the logistic regression are shown in Table 5. For all measures except for the offer of a canteen with healthy food, gender influences could be identified. Gender had a negative effect on the response to offered company sports, where the probability of nonparticipation was higher for women (OR = 1.46, 95% CI = 1.16–1.84, p = 0.001). In the case of the other offers, there was a positive influence: the probability of women not participating in the measures was lower (OR = between 0.76 and 0.56, 95% CI between 0.49 and 0.99, p = <0.001 and 0.038).

**TABLE 5:** Results of a logistic regression on different workplace health promotion measures (back health, company sport, canteen with healthy lunch, information/consulting on healthy nutrition, stress management, discussion/working groups on health problems, help in coping with bullying and conflicts, financial subsidy for health offers) with sociodemographic (gender, age), socioeconomic (socioeconomic status, subjective social status), health-related (subjective health status, attention to health, body mass index, smoking, alcohol, sport, nutrition), company-related (number of employees, business sector, professional status, working hours), social support and life satisfaction variables using data from the Robert Koch Institute (GEDA study 2014/2015-EHIS, Berlin 2017, n = 7,912)^a^.

	Healthy lunch (n = 3,500)		Back health (n = 2,874)		Company sport (n = 2,570)	
	OR (95% CI)	p-value	OR (95% CI)	p-value	OR (95% CI)	p-value
Gender						
Men	Ref.		Ref.		Ref.
Women	1.12 (0.93–1.33)	0.227	0.61 (0.49–0.77)	<0.001	1.46 (1.16–1.84)	0.001
Age						
18–29 years	Ref.		Ref.		Ref.
30–44 years	1.32 (1.03–1.69)	0.026	0.84 (0.61–1.14)	0.257	1.52 (1.14–2.02)	0.005
45–64 years	1.67 (1.32–2.12)	<0.001	0.83 (0.62–1.13)	0.240	1.92 (1.44–2.55)	<0.001
Socioeconomic Status (SES)						
Low	Ref.		Ref.		Ref.
Middle	1.31 (0.95–1.79)	0.096	1.27 (0.84–1.90)	0.258	1.08 (0.67–1.76)	0.750
High	0.87 (0.62–1.24)	0.447	1.68 (1.08–2.60)	0.021	1.47 (0.88–2.46)	0.138
Subjective social status						
Lower-middle	Ref.		Ref.		Ref.
Higher-upper	0.76 (0.65–0.90)	0.001	1.18 (0.96–1.45)	0.113	1.02 (0.82–1.27)	0.863
Subjective Health Status						
Poor-moderate	Ref.		Ref.		Ref.
Good-very good	0.99 (0.81–1.20)	0.898	1.03 (0.80–1.33)	0.824	0.61 (0.45–0.83)	0.002
Attention to health						
Little-not at all	Ref.		Ref.		Ref.
Strong-very strong	0.85 (0.73–0.99)	0.048	0.72 (0.59–0.88)	0.001	0.71 (0.58–0.88)	0.001
BMI						
Underweight	Ref.		Ref.		Ref.
Normal weight	0.90 (0.48–1.64)	0.706	1.90 (0.90–3.98)	0.087	0.78 (0.33–1.84)	0.565
Overweight	0.93 (0.50–1.72)	0.812	1.86 (0.88–3.92)	0.106	0.81 (0.34–1.95)	0.640
Obesity	0.92 (0.49–1.74)	0.808	1.96 (0.90–4.22)	0.087	0.76 (0.31–1.87)	0.548
Smoking						
Yes	Ref.		Ref.		Ref.
No	0.97 (0.81–1.15)	0.699	0.92 (0.73–1.15)	0.445	0.94 (0.75–1.19)	0.606
Alcohol						
Yes	Ref.		Ref.		Ref.
No	0.97 (0.81–1.15)	0.699	1.10 (0.87–1.40)	0.431	1.14 (0.88–1.48)	0.321
Sport per week						
No - little sport	Ref.		Ref.		Ref.
Much - daily sport	1.15 (0.96–1.39)	0.134	1.08 (0.84–1.38)	0.562	0.62 (0.49–0.78)	<0.001
Nutrition						
Unhealthy nutrition	Ref.		Ref.		Ref.
Healthy nutrition	0.99 (0.84–1.16)	0.875	0.96 (0.78–1.17)	0.699	0.84 (0.68–1.04)	0.111
Number of employees in the company						
0–10	Ref.		Ref.		Ref.
11–19	1.64 (0.90–2.99)	0.108	0.92 (0.54–1.57)	0.755	0.76 (0.38–1.51)	0.435
20–49	2.48 (1.42–4.33)	0.001	1.08 (0.65–1.81)	0.746	0.76 (0.40–1.46)	0.416
50+	2.58 (1.57–4.24)	<0.001	1.87 (1.22–2.87)	0.004	1.20 (0.69–2.10)	0.521
Business sector						
Manufacturing and processing industries	Ref.		Ref.		Ref.
Service sector	0.83 (0.68–1.03)	0.093	1.03 (0.80–1.35)	0.821	1.06 (0.80–1.41)	0.676
Public service	1.26 (1.03–1.55)	0.024	1.15 (0.89–1.49)	0.289	0.90 (0.69–1.19)	0.462
Others	0.54 (0.30–0.98)	0.044	0.94 (0.53–1.65)	0.822	0.54 (0.30–0.96)	0.035
Professional position						
Employee	Ref.		Ref.		Ref.
Worker	1.53 (1.19–1.97)	<0.001	1.20 (0.79–1.59)	0.527	1.15 (0.77–1.72)	0.485
Officer	1.43 (1.12–1.83)	0.004	0.86 (0.64–1.16)	0.315	0.34 (0.26–0.45)	<0.001
Trainee	1.16 (0.75–1.78)	0.513	1.37 (0.74–2.56)	0.315	1.36 (0.91–2.92)	0.099
Working hours						
Full-time	Ref.		Ref.		Ref.
Part-time	1.39 (1.14–1.70)	0.001	1.66 (1.28–2.16)	<0.001	1.54 (1.14–2.08)	0.004
Marginally employed	0.86 (0.47–1.56)	0.613	1.65 (0.72–3.81)	0.238	0.47 (0.18–1.19)	0.110
Partial retirement	1.26 (0.69–2.29)	0.456	1.05 (0.48–2.29)	0.913	1.90 (0.54–6.75)	0.321
Social support						
Low	Ref.		Ref.		Ref.
Middle	0.74 (0.59–0.92)	0.008	0.86 (0.64–1.15)	0.294	0.66 (0.47–0.91)	0.011
High	0.83 (0.65–1.07)	0.150	0.92 (0.67–1.26)	0.595	0.58 (0.41–0.83)	0.003
Life satisfaction						
Not at all-rather	Ref.		Ref.		Ref.
satisfied-completely satisfied	0.87 (0.67–1.14)	0.301	0.88 (0.61–1.27)	0.493	1.08 (0.73–1.61)	0.699

	Stress management (n = 2,465)		Info/Consulting on nutrition (n = 2,275)		Help against bullying (n = 2,134)	
	OR (95% CI)	p-value	OR (95% CI)	p-value	OR (95% CI)	p-value
Gender						
Men	Ref.		Ref.		Ref.
Women	0.66 (0.53–0.82)	<0.001	0.69 (0.56–0.85)	<0.001	0.56 (0.42–0.75)	<0.001
Age						
18–29 years	Ref.		Ref.		Ref.
30–44 years	1.29 (0.95–1.75)	0.104	1.21 (0.89–1.64)	0.230	1.50 (1.01–2.33)	0.047
45–64 years	1.01 (0.75–1.36)	0.951	0.77 (0.57–1.03)	0.082	1.55 (1.06–2.27)	0.025
Socioeconomic status (SES)						
Low	Ref.		Ref.		Ref.
Middle	1.54 (0.96–2.46)	0.071	0.92 (0.62–1.37)	0.692	2.22 (1.33–3.69)	0.002
High	1.64 (1.00–2.68)	0.049	0.85 (0.56–1.30)	0.459	2.67 (1.53–4.68)	<0.001
Subjective social status						
Lower-middle	Ref.		Ref.		Ref.
Higher-upper	1.03 (0.84–1.26)	0.797	1.06 (0.87–1.29)	0.574	1.04 (0.79–1.37)	0.773
Subjective Health Status						
Poor-moderate	Ref.		Ref.		Ref.
Good-very good	1.32 (1.02–1.71)	0.035	1.06 (0.83–1.36)	0.632	1.47 (1.07–2.02)	0.019
Attention to health						
Little-not at all	Ref.		Ref.		Ref.
Strong-very strong	0.69 (0.57–0.84)	<0.001	0.70 (0.58–0.84)	<0.001	0.68 (0.52–0.88)	0.004
BMI						
Underweight	Ref.		Ref.		Ref.
Normal weight	0.80 (0.36–1.81)	0.594	1.42 (0.70–2.91)	0.335	1.11 (0.47–2.61)	0.815
Overweight	0.73 (0.32–1.67)	0.457	1.40 (0.67–2.88)	0.376	1.11 (0.46–2.67)	0.819
Obesity	0.57 (0.24–1.32)	0.188	1.30 (0.62–2.74)	0.489	1.07 (0.41–2.56)	0.956
Smoking						
Yes	Ref.		Ref.		Ref.
No	1.34 (1.08–1.67)	0.008	1.22 (0.99–1.50)	0.055	1.16 (0.87–1.55)	0.307
Alcohol						
Yes	Ref.		Ref.		Ref.
No	0.88 (0.70–1.12)	0.297	1.01 (0.80–1.28)	0.912	1.06 (0.78–1.44)	0.703
Sport per week						
No - little sport	Ref.		Ref.		Ref.
Much - daily sport	0.80 (0.64–1.01)	0.063	0.78 (0.63–0.98)	0.033	0.89 (0.66–1.21)	0.454
Nutrition						
Unhealthy nutrition	Ref.		Ref.		Ref.
Healthy nutrition	0.89 (0.72–1.09)	0.248	0.80 (0.66–0.98)	0.028	0.91 (0.69–1.20)	0.495
Number of employees in the company						
0–10	Ref.		Ref.		Ref.
11–19	1.17 (0.68–1.99)	0.570	1.23 (0.76–1.99)	0.400	1.07 (0.59–1.93)	0.836
20–49	0.65 (0.39–1.06)	0.083	1.69 (1.07–2.67)	0.023	1.11 (0.62–1.97)	0.734
50+	1.46 (0.95–2.24)	0.085	2.35 (1.63–3.40)	<0.001	2.08 (1.29–3.35)	0.003
Business sector						
Manufacturing and processing industries	Ref.		Ref.		Ref.
Service sector	1.01 (0.76–1.35)	0.921	1.24 (0.97–1.59)	0.092	0.86 (0.57–1.30)	0.473
Public service	1.03 (0.78–1.34)	0.859	1.43 (1.12–1.84)	0.004	0.63 (0.43–0.92)	0.016
Others	1.14 (0.65–2.00)	0.650	1.82 (1.06–3.14)	0.030	0.41 (0.22–0.78)	0.006
Professional position						
Employee	Ref.		Ref.		Ref.
Worker	1.93 (1.16–3.20)	0.011	0.99 (0.71–1.39)	0.962	0.81 (0.48–1.36)	0.429
Officer	0.93 (0.71–1.21)	0.564	1.05 (0.78–1.40)	0.771	1.10 (0.77–1.58)	0.591
Trainee	0.71 (0.41–1.24)	0.231	0.71 (0.41–1.21)	0.208	0.56 (0.29–1.08)	0.084
Working hours						
Full-time	Ref.		Ref.		Ref.
Part-time	0.96 (0.76–1.22)	0.762	0.97 (0.76–1.24)	0.827	1.05 (0.77–1.45)	0.751
Marginally employed	0.81 (0.37–1.74)	0.582	1.08 (0.53–2.18)	0.842	0.91 (0.35–2.36)	0.847
Partial retirement	0.60 (0.28–1.28)	0.186	0.83 (0.39–1.75)	0.619	0.64 (0.27–1.56)	0.329
Social support						
Low	Ref.		Ref.		Ref.
Middle	1.09 (0.82–1.45)	0.563	0.86 (0.64–1.16)	0.319	1.07 (0.71–1.62)	0.749
High	1.11 (0.82–1.51)	0.501	0.90 (0.66–1.24)	0.513	0.80 (0.52–1.24)	0.323
Life satisfaction						
Not at all-rather	Ref.		Ref.		Ref.
Satisfied-completely satisfied	1.15 (0.81–1.64)	0.422	1.04 (0.73–1.50)	0.826	1.52 (0.98–2.36)	0.064

	Financial allowance (n = 1,690)		Discussion/Working group (n = 1,137)	
	OR (95% CI)	p value	OR (95% CI)	p value
Gender				
Men	Ref.		Ref.
Women	0.76 (0.58-0.99)	0.038	0.81 (0.59-1.10)	0.177
Age				
18-29 years	Ref.		Ref.
30-44 years	1.36 (0.97–1.92)	0.078	1.06 (0.68–1.65)	0.796
45-64 years	1.24 (0.89–1.74)	0.199	0.86 (0.56–1.31)	0.478
Socioeconomic status (SES)				
Low	Ref.		Ref.
Middle	1.08 (0.66–1.77)	0.757	0.84 (0.50–1.43)	0.528
High	1.03 (0.60–1.76)	0.913	1.20 (0.68–2.14)	0.535
Subjective social status				
Lower-middle	Ref.		Ref.
Higher-upper	1.27 (0.99–1.63)	0.061	1.33 (0.99–1.78)	0.054
Subjective Health Status				
Poor-moderate	Ref.		Ref.
Good-very good	0.77 (0.56–1.07)	0.120	1.39 (0.98–1.97)	0.064
Attention to health				
Little-not at all	Ref.		Ref.
Strong-very strong	0.78 (0.61–0.98)	0.033	0.77 (0.58–1.02)	0.068
BMI				
Underweight	Ref.		Ref.
Normal weight	1.03 (0.39–2.73)	0.960	0.82 (0.21–3.18)	0.776
Overweight	1.14 (0.42–3.07)	0.799	0.74 (0.19–2.92)	0.670
Obesity	0.93 (0.34–2.59)	0.893	0.77 (0.19–3.08)	0.708
Smoking				
Yes	Ref.		Ref.
No	0.86 (0.67–1.14)	0.322	1.34 (0.99–1.82)	0.056
Alcohol				
Yes	Ref.		Ref.
No	1.28 (0.95–1.73)	0.106	0.75 (0.54–1.05)	0.091
Sport per week				
No - little sport	Ref.		Ref.
Much - daily sport	0.89 (0.67–1.18)	0.421	1.01 (0.72–1.41)	0.964
Nutrition				
Unhealthy nutrition	Ref.		Ref.
Healthy nutrition	0.72 (0.57–0.93)	0.010	0.91 (0.68–1.22)	0.529
Number of employees in the company				
0-10	Ref.		Ref.
11-19	1.13 (0.60–2.16)	0.700	1.66 (0.77–3.55)	0.195
20-49	1.57 (0.84–2.93)	0.159	1.34 (0.64–2.84)	0.441
50+	2.14 (1.27–3.62)	0.004	2.98 (1.60–5.52)	<0.001
Business sector				
Manufacturing and processing industries	Ref.		Ref.
Service sector	0.94 (0.70–1.25)	0.661	1.27 (0.87–1.85)	0.217
Public service	0.97 (0.72–1.31)	0.823	0.95 (0.66–1.37)	0.791
Others	0.48 (0.25–0.90)	0.023	2.26 (0.90–5.67)	0.081
Professional position				
Employee	Ref.		Ref.
Worker	0.96 (0.63–1.46)	0.856	0.78 (0.49–1.24)	0.292
Officer	1.04 (0.64–1.68)	0.876	1.16 (0.77–1.76)	0.477
Trainee	1.53 (0.73–3.20)	0.257	2.76 (1.04–7.35)	0.042
Working hours				
Full-time	Ref.		Ref.
Part-time	1.23 (0.91–1.67)	0.178	0.85 (0.59–1.22)	0.374
Marginally employed	1.04 (0.37–2.91)	0.946	0.52 (0.16–1.70)	0.280
Partial retirement	1.69 (0.53–5.42)	0.376	0.57 (0.19–1.72)	0.314
Social support				
Low	Ref.		Ref.
Middle	1.09 (0.76–1.55)	0.653	0.65 (0.42–1.01)	0.054
High	1.16 (0.79–1.71)	0.445	0.65 (0.41–1.04)	0.071
Life satisfaction				
Not at all-rather	Ref.		Ref.
satisfied-completely satisfied	0.93 (0.58–1.50)	0.765	0.90 (0.53–1.54)	0.705

^a^ In the next tables, the following abbreviations are used: OR, odds ratio; CI, 95% Confidence Interval, p = Significance, Ref. = reference group.

Age was identified as a negative factor for three measures: company sports, canteen with healthy food and help against bullying. The probability of nonparticipation increased with age.

In terms of SES, three offers showed an increased probability of nonparticipation of individuals with a medium or high SES (back health, stress management, help against bullying).

There was virtually no influence of individuals’ subjective perception of their situation in society, except for one offer: Assignment to the “higher to upper social class” decreased the probability of nonparticipation in healthy lunch (OR = 0.76, 95% CI = 0.65–0.90, p = 0.001).

There was an increase in the probability of nonparticipation associated with subjectively perceived health status for two measures (stress management, help against bullying) when the state of health was described as “good - very good.” The opposite effect was observed in the case of company sports.

A broad influence could also be seen in the attentiveness to health: seven measures showed that the probability of not participating in the offer decreased if the individual was very attentive to their health.

Regarding health lifestyle indicators, weight and alcohol consumption were found to have no influence. The smoking indicator showed that nonsmokers were less likely to participate in the stress management program than smokers were. The sport activity indicator presented an influence on two measures (company sport, info/consulting nutrition): those who had many sports activities per week tended not to participate. The same effect was observed for the indicator for nutrition in two measures (info/consulting nutrition, financial support for sports activities).

The size of the company also had an impact on nonparticipation: the larger the company was, the greater the probability of nonparticipation.

Business sector may also influence nonparticipation. The logistic regression showed both an increase and a decrease in the probability of nonparticipation. The probability of nonparticipation was lower in the sector “others” for the offers company sports, healthy lunch and financial support for sports activities. This is also evident for the offer help against bullying in the sector “public service” compared to the industrial sector. While nonparticipation in the sector “public service” was higher in the offer healthy canteen, this also applies to the offer information/advice on healthy nutrition in the sector “others” compared to the industrial sector.

Professional position could also have an influence in both directions. There were only isolated influences among workers, officers and trainees. Trainees had a higher probability of not participating in offers compared to employees for all measures except for the company sport measure.

Regarding hours worked, nonparticipation was higher among part-time employees compared to full-time for the three measures back health, company sport and healthy lunch.

A medium to high level of social support compared to a low level led to a lower probability of nonparticipation for the two measures company sport and healthy lunch.

Life satisfaction did not have any significant influence in the logistic regression.

The factors of gender, attentiveness to health and company size have a strong influence, followed by company size and occupational position. Age, SES, subjectively perceived health status and working hours also had significant influence. The health lifestyle indicators showed only low significance.

The most influential factors were found for offering company sports (nine factors), followed by offering a canteen with healthy food (eight factors) and offering help against bullying (seven factors). For all offers except the offer of discussion or working groups on health problems, both positive and negative effects on nonparticipation were found. The studied factors influence the probability of nonparticipation to different degrees and in complex ways. In Supplementary Table S1 (to be found in the Supplementary Material), the effects of the variables examined are summarised.

## Discussion

The results of the presented study confirm or supplement some findings of other studies but also highlight differences and give new insights on the basis of a representative sample of Germany and therefore fill the gap in previous research.

The factors previously considered in studies on participation - gender, age, SES, company size, sector, employment, subjective health status, health awareness [6, 20–22, 28, 30] - also showed an influence on nonparticipation, even for WHP measures not previously investigated. Subjective social status, sporting activity, nutrition and general social support are new significant factors which contribute to nonparticipation in WHP-measures.

There are differences in usage behaviour, which are particularly evident in relation to gender. With the exception of company sports, there was significant less nonparticipation among women than among men. Previous studies show that this is possibly due to the fact that women pay more attention to their health and are generally more likely to exploit preventive health services and engage in less risky activities [21, 22]. Depending on the design, objective and availability of a health promotion measure, it is more likely to appeal to men or to women [20–22]. According to Beck et al. (2016), WHP measures generally seem to have a stronger orientation towards men [20]. If company sport programmes are indeed more designed for men, including things like advertising, rooms, type of sport offered (which wasn’t asked for in the GEDA study), this could indeed lead to less participation of women. However, there are numerous other aspects that might play a role, for example, unlike other WHP measures sport takes dedicated time which might interfere with time demands of women from care work.

Gender differences (higher (non-)participation sometimes among women sometimes among men) also depend on the study context, so different results could be due to different research settings [20–23, 30].

In addition to gender, there are also differences between the age groups. As in other studies [22, 30], we found that probability of nonparticipation increased with age for half of the tested measures. This could be due to the fact not all offers meet the needs of older employees or are adapted to them [31]. Considering higher nonparticipation in measures together with decreasing work ability due to age [32], occupational health management should consider this aspect more closely: The well-researched theory of selective optimization with compensation, as a model of “successful ageing,” might be a good starting point to offer measures which are more than just a transfer of the concepts for the “older colleagues” [33].

Similar to other studies [21, 22], part-time employment increases the likelihood of nonparticipation. The shorter presence at the workplace and thus also the potentially lower awareness of offers and time for measures could be the reason for this effect.

The business sector [6, 20, 22, 28] and professional position also have a significant influence on participation behaviour. These aspects show once again that WHP measures must always be considered in the context of their application, as the needs (e.g., in the respective sectors) are very different. Specific programmes are required depending on the requirements of the work activity. Therefore, it will not be sufficient for a company to offer WHP measures in general. WHP always should consider the specific requirements of the jobs and departments. Due to the limitations of the GEDA questionnaire, this aspect cannot be analysed in more detail and will require more specific consideration in future research.

Large companies have more opportunities to offer and implement WHP measures, presumably due to their better financial and personnel resources [34, 35]. Nevertheless, nonparticipation increases with the size of the company, which is also a result of other studies [6, 22]. It is hypothesised here and elsewhere that anonymity could play a relevant role in large companies. Communication channels in large companies are often longer and run through several departments, which might leave employees unaware of available offers, unable to assess their relevance to their work and health, or unsure how to incorporate them into their working schedule if it comes from a “far-away” department.

In this study, the indicators for weight, smoking, alcohol, exercise and nutrition were also taken into account. The fact that there were hardly any influences here could be due to the fact that they are based on subjective assessments. The answers could therefore be biased, and it cannot be ruled out that the results reflect socially desirable responses rather than reality in terms of external validity [21]. It is therefore questionable to what extent these indicators should continue to be considered in the future, especially as weight continues to be classified using the BMI, which has already been criticised [36]. Nonparticipation decreased with a healthy nutrition and a high level of physical activity. Theories and studies on health behaviour support the fact that individual actions can generally have a positive effect on health and thus positively influence health behaviour and the use of prevention measures [22, 23, 37]. Based on the goal of health-promoting offers that employees feel and stay healthy, consideration should be given to how and with what measures especially people with a low level of health awareness, an unhealthy diet and little exercise can be addressed.

Social support of friends, family and neighbours only had a limited influence on nonparticipation in our study. Nöhammer et al. (2023) [19] instead show a completely different influence of social support: motivation and support from colleagues and superiors can have a positive effect on participation behaviour. But there is a fine line between motivation and perceived pressure, which has an inhibiting effect.

With regard to the reasons for nonparticipation, future research should look more closely at gender and, due to demographic change, also at older employees. The SES should also be considered. In general, studies show that SES has a significant influence without exception, and various research results have revealed coherence, especially between education and health [21–23]. It should therefore also be further investigated whether the WHP measures address all levels of the SES.

In the present study, we found that factors known to influence participation in WHP measures also seem to play a role in nonparticipation, and we identified some new factors.

Possible limitations of the study must also be considered. The attempt to include as many possible influencing factors in the present analysis increased our model of regression. Significance and explained variance can be influenced by the number of independent variables. Further possible correlations and influences could not be recognised. At this point, reference should be made to Hermann et al. (2021), who list further limitations regarding such studies [21]. For example, answers were based on a self-assessment, which could lead to distortion by a false self-perception or perceived social undesirability. In the GEDA questionnaire two questions were asked referring to WHP measures, which also limits the scope of the study. There can also be a loss of information when summarising answer options. Since this work is intended to consider as many potential influences as possible in a broad spectrum, this loss of information seems to be acceptable from our point of view. Finally, the age of the data also represents a limitation, as the world of work and society have changed significantly since 2014/2015. It could be assumed, for example, that the greater acceptance of mental health problems on a societal level also means a higher supply and greater acceptance of WHP. As no other population-representative data is available and the variables analysed are presumably less affected by these changes, our results nevertheless provide initial relevant insights. However, a comparison with current or longitudinal data would be necessary to prove to what extent the results are still valid (especially after the COVID pandemic) or influenced by a cohort effect. The cohort effect would show possible differences between groups that can be attributed to various social and environmental changes between generations. Contrary to the assumption of more WHP measures due to social change, it was for example found that due to the COVID pandemic, WHP measures no longer have such a high priority in companies [38]. Further research is therefore needed about how WHP offers have changed and whether this has an impact on usage behaviour.

### Conclusion

Even though an increasing number of companies are introducing WHP measures, not all employees participate. The factors influencing nonparticipation can be considered at different levels. Demographic factors, such as age and gender, have a very strong influence, with age increasing the probability of nonparticipation. Regarding demographic changes in modern Western societies, a stronger consideration of older employees is becoming increasingly important. It can be confirmed that men are less likely to participate in the offers, and generally poor health behaviour and negative health attitudes increase the probability of nonparticipation. This topic also seems to be related to (un)equitable access to and participation in healthcare services [39].

Regarding gender, health behaviour and attitudes, WHP measures should be evaluated in terms of design, objectives and availability. Also, offers should be accessible to part-time employees or appeal to employees with lower perceived socioeconomic or worse health status.

The size of the company plays a major role in nonparticipation of WHP measures. Not only do smaller companies need to be better informed about the possibilities and benefits of introducing WHP measures, but also large companies should be made aware of the reasons why many of their employees do not exploit their offers. Especially in the present, when the labour market and conditions are rapidly changing due to globalisation and demographic change, it is becoming more important that employees not only feel well and stay healthy but also feel supported in doing so by their company. It is important to pay more attention to the influences and reasons for nonparticipation in such programmes. Therefore, more attention should be given to the target group to increase rates of participation.

Further research is needed to determine whether the influencing factors presented here are actually the sole reason for nonparticipation or whether there are other barriers, such as lack of time or local accessibility.

The present study was able to identify a number of possible influencing factors. However, there are still unanswered questions that do not emerge from the questionnaire and require further – qualitative - investigations: how often, when and where were the respective measures offered? These aspects can also have an influence on nonparticipation, as previous small-scale studies, mainly abroad, have shown [8, 17–19, 31]. The present study shows that there is a research gap, particularly in the area of nonparticipation and its reasons.
