# Supplementary material for: A Representative Analysis of Nonparticipation in Workplace Health Promotion in Germany Using Multivariable Methods
Source: Int J Public Health. 2024 Dec 5;69:1607261. doi: 10.3389/ijph.2024.1607261 (PMC11655221; doi:10.3389/ijph.2024.1607261)
Supplement: Supplementary file 1 [file Table1.pdf]

Supplement Material:

Table S1: Effects of sociodemographic (gender, age), socioeconomic (socioeconomic status, subjective social status), health-related (subjective health status, attentiveness to health, body mass index, smoking, alcohol, sport, diet), company-related (number of employees, industry, occupational status, working hours), social support and life satisfaction variables on nonparticipation using data from the Robert Koch Institute (GEDA study 2014/2015-EHIS, Berlin 2017, n = 7,912)

Nonparticipation increases (+), nonparticipation decreases (-), and no effect (xx).

**Variable**	**Effect**	**Variable**	**Effect**
Gender (Ref. Men)	+/-	Sport per week (Ref. No-little sport)	-
Age (Ref. 18-29 years)	+	Nutrition (Ref. Unhealthy nutrition)	-
Socioeconomic status (SES) (Ref. Low)	+	Number of employees in the company (Ref. 0-10)	+
Subjective social status (Ref. Lower-middle)	-	Business sector (Ref. Manufacturing and processing industries)	+/-
Subjective health status (Ref. Poor-moderate)	+/-	Professional position (Ref. Employee)	+/-
Attention to health (Ref. Little-not at all)	-	Working hours (Ref. Full-time)	+
BMI (Ref. Underweight)	xx	Social support (Ref. Low)	-
Smoking (Ref. Yes)	+	Life satisfaction (Ref. Not at all-rather)	xx
Alcohol (Ref. Yes)	xx		
